# Monte Carlo simulation of expected outcomes with the AcrySof^® ^toric intraocular lens

**DOI:** 10.1186/1471-2415-8-22

**Published:** 2008-10-27

**Authors:** Warren Hill, Richard Potvin

**Affiliations:** 1 East Valley Ophthalmology, 5620 East Broadway Road, Mesa, Arizona, 85206, USA; 2 Alcon Laboratories, Inc., 6201 S. Freeway, Fort Worth, Texas, 76134, USA

## Abstract

**Background:**

To use a Monte Carlo simulation to predict postoperative results with the AcrySof^® ^Toric lens, evaluating the likelihood of over- or under-correction using various toric lens selection criteria.

**Methods:**

Keratometric data were obtained from a large patient population with preoperative corneal astigmatism <= 2.50D (2,000 eyes). The probability distributions for toric marking accuracy, surgically induced astigmatism and lens rotation were estimated using available data. Anticipated residual astigmatism was calculated using a Monte Carlo simulation under two different lens selection scenarios.

**Results:**

This simulation demonstrated that random errors in alignment, surgically induced astigmatism and lens rotation slightly reduced the overall effect of the toric lens. Residual astigmatism was statistically significantly higher under the simulation of surgery relative to an exact calculation (p < 0.05). The simulation also demonstrated that more aggressive lens selection criteria could produce clinically significant reductions in residual astigmatism in a high percentage of patients.

**Conclusion:**

Monte Carlo simulation suggests that surgical variability and lens orientation/rotation variability may combine to produce small reductions in the correction achieved with the AcrySof^® ^Toric^® ^IOL. Adopting more aggressive lens selection criteria may yield significantly lower residual astigmatism values for many patients, with negligible overcorrections. Surgeons are encouraged to evaluate their AcrySof^® ^Toric^® ^outcomes to determine if they should modify their individual lens selection criteria, or their default surgically induced astigmatism value, to benefit their patients.

## Background

Continued advances in small-incision phacoemulsification have increased the stability and predictability of cataract surgery, reducing healing time and intraoperative/postoperative complications. For normal eyes, modern cataract surgery can typically provide a refractive correction that is often within 0.5D of the targeted spherical correction [1-4].

Advances in the ability to correct the refractive errors of cataract patients have also been made, particularly with regard to astigmatism. Corneal astigmatism is often reduced by the use of peripheral corneal relaxing incisions, but the introduction of toric intraocular lenses now provides an opportunity to more precisely reduce or eliminate a patient's astigmatism, particularly if consideration of the induced astigmatism from the surgical incision is included to calculate the expected postoperative corneal astigmatism [5-8].

The AcrySof Toric lens is used with a new method for correcting corneal astigmatism in pseudophakic eyes. This lens is implanted in conjunction with a Toric Lens Calculator, which uses surgeon-provided keratometry and surgically induced astigmatism data to select the most appropriate toric lens and calculate the optimal angle of placement (, Alcon Laboratories, Inc.). Three different powers at the lens plane provide nominal corneal plane correction of 1.03D, 1.55D and 2.06D of astigmatism in a variety of spherical powers (Product Information, AcrySof Toric, Alcon Laboratories, Inc.).

The success of a toric lens hinges on accurate and stable correction. With regard to accuracy, the surgically induced astigmatism must be taken into account. The lens must be implanted in precise alignment with the required axis of correction; this is usually achieved using a corneal marker to intraoperatively identify the correct lens orientation. With regard to stability, the lens must maintain its intended orientation over time. This latter element is key to the success of the lens over the long term [9,10].

The challenge with regard to accurate and stable correction is that ophthalmic surgery is, by its very nature, a variable procedure. Surgically induced astigmatism, while it can be quite low on average, will vary from eye to eye and surgeon to surgeon, and can have a significant effect on outcomes [11,12]. The process of marking the cornea to properly align the toric lens may also be less than perfect and the axis alignment achieved at the time of surgery may not be exact. Individual lenses may also rotate during the postoperative period. All of these rather small errors in alignment and stability will typically combine to reduce the potential effectiveness of any toric lens; the more these errors are brought under control, the lower the potential effect. In general, each 1 degree error in lens alignment will reduce the effectiveness of the astigmatism correction by 3.33%.

The current version of the Alcon toric calculator provides the surgeon a lens recommendation that is based on avoiding any overcorrection, as patients are believed more tolerant of low levels of astigmatism along their original axis than they are of astigmatism that is orthogonal to it. However, given the challenges in lens orientation and stability, it may be that this approach is too conservative to be practical. For instance, a patient with 1.03D of astigmatism is recognized as a candidate for the 1.03 diopter toric lens (T3). However, a patient with 0.80D may not be. This is because this patient would, in the case of exact correction, be left with 0.80D of astigmatism if a spherical lens were used but if the T3 lens were to be used this same patient might be left with 0.23D of astigmatism in an orthogonal meridian, which is deemed an undesirable result. The practical limitation here is that the analysis presumes a perfect correction. Because astigmatic correction is directional, a deviation from the desired orientation and stability of the toric lens will reduce the effect of the lens in the desired direction and introduce a corresponding change in net axis of astigmatism; the likelihood of an overcorrection of astigmatism is significantly lower when this occurs.

One might then argue that such a patient would have been better off with a T3 correction rather than the spherical correction recommended by the AcrySof toric IOL calculator.

The mathematical exercise in this paper was to assume that the factors involved in toric lens implantation are slightly variable, and to predict the likely effects of that variability using a Monte Carlo simulation with different lens selection criteria.

## Methods

The method of a Monte Carlo simulation as used here presumes that independent random events when occurring in sequence can be combined to predict real world results if the probability of each event can be reasonably determined. The random variables used in this simulation included surgically induced astigmatism, corneal marking, and lens rotation. The fixed variables were the sample of eyes with preoperative keratometry data, the presumed surgically induced astigmatism for use in the Acrysof^® ^Toric^® ^IOL calculator and the selection criteria for the toric lens to use. Each is described below.

The simulation values below are based on a given known (or presumed) distribution of results. A cumulative distribution function for each variable can be constructed using the assumptions below. A random number generator is then used to generate 2,000 random numbers between 0 and 1.0 and the related variable value is determined by this function. For instance presume the distribution of lens rotation is such that 25% of lenses rotated 2 degrees, 25% rotated 5 degrees and 50% rotated 10 degrees. The 2,000 random numbers are generated. If the value generated is less than 0.25 then the rotation assigned is 2, if between 0.25 and 0.50 the rotation assigned is 5 and anything higher than 0.50 is assigned a value of 10. The actual distribution functions here are more complex than this but the method is the same.

There are, of course, other variables that may affect the surgery. The manufacturing process may have some inherent variability, but standards do limit this; relevant data are not publicly available and were not included here. The preoperative keratometry readings would also have some variability. Actual IOL placement relative to the reference markings on the cornea would have an additive effect to the lens rotation error estimated here. The lens astigmatism power is specified at the IOL plane and the corneal plane effect is presumed on the basis of nominal biometry; variability in axial length will change the astigmatism correction at the corneal plane. All of these secondary variables were assumed to be less important than the three included in the current model. Their exclusion does not invalidate the simulation model; it merely simplifies the model but might also mean that the model may predict a slightly better result, or predict slightly less variability, than might be achieved in practice.

Surgically induced astigmatism was estimated, presuming a mean value of 0.50D. This is consistent with the mean surgically induced astigmatism calculated from data collected by WH on a web site that allowed surgeons to calculate their individual surgically induced astigmatism by incision type and location, with the aim of improving the results from the on-line toric calculator mentioned above. The mean surgically induced astigmatism value for a total of 92 surgeries with 2.2 mm incisions was 0.6 D. These and other aggregate data from the web site in question will be reported in a more extensive paper in future.

Evidence suggests that this average value is reasonable for small incisions, but that results can vary in both magnitude and axis. For the purposes of this simulation the magnitude was presumed to be truncated normal distribution with a mean of 0.50D and a standard deviation of 0.12D, with a minimum value of 0.0D and a maximum value of 1.0D. The axis of the surgically induced astigmatism was presumed to be a truncated normal distribution around a mean of 90 degrees (the incision flattens the meridian that is cut, equivalent to a relative steepening of the orthogonal meridian [13]) with a standard deviation of 2 degrees, a minimum of 80 degrees and a maximum of 100 degrees.

Lens marking was also presumed to be variable because head tilt and mark size make it difficult to ensure perfect accuracy. The lens marking was presumed to be a truncated normal distributed around 0 degrees with a standard deviation of 1.5 degrees, a minimum of -5 degrees and a maximum of 5 degrees. This suggests that 95% of markings would be within ± 3 degrees of intended.

Finally, lens rotation was estimated using the data from Alcon provided in the lens labeling. The average rotation reported in the Directions For Use was less than 5 degrees for the majority of patients and similar results have been reported in the literature [14-16]. Using the data collected in the Alcon clinical study for the FDA a cumulative distribution curve for lens rotation was constructed. Using 2,000 random numbers a table of relative rotations was prepared; this is a well-established method for creating a sample from a cumulative distribution curve.

Sample data for keratometric astigmatism were obtained for a large patient population with preoperative corneal astigmatism <= 2.50D (2,000 eyes). The AcrySof^® ^Toric^® ^calculator  was used to determine the resultant corneal astigmatism, presuming a surgically induced astigmatism value of 0.50D at 90 degrees (temporal incision). On the basis of this resultant corneal astigmatism, toric lenses were selected according to two different criteria. The first was the initial set of criteria provided by Alcon in their toric calculator; these criteria were set to minimize the likelihood of any overcorrection. The second set of criteria was designed to raise the likelihood of overcorrection slightly but to lower the likelihood of residual astigmatism in the eye. These criteria are shown in Table 1. The results from the available corneal astigmatism data and the toric lens calculator produced a table of preoperative corneal cylinder data with two possible lens selections.

**Table 1:** Lens selection criteria based on Resultant Corneal Astigmatism

(post-incision, based on the toric calculator)			
	T3 (1.03D)*	**T4 (1.55)**	**T5 (2.06)**
**Current Selection**	>= 1.03D	>= 1.55D	>= 2.10D
**Aggressive Selection**	>= 0.75D	>= 1.35D	>= 1.90D

* nominal lens astigmatic power at the corneal plane

**Figure 1:**
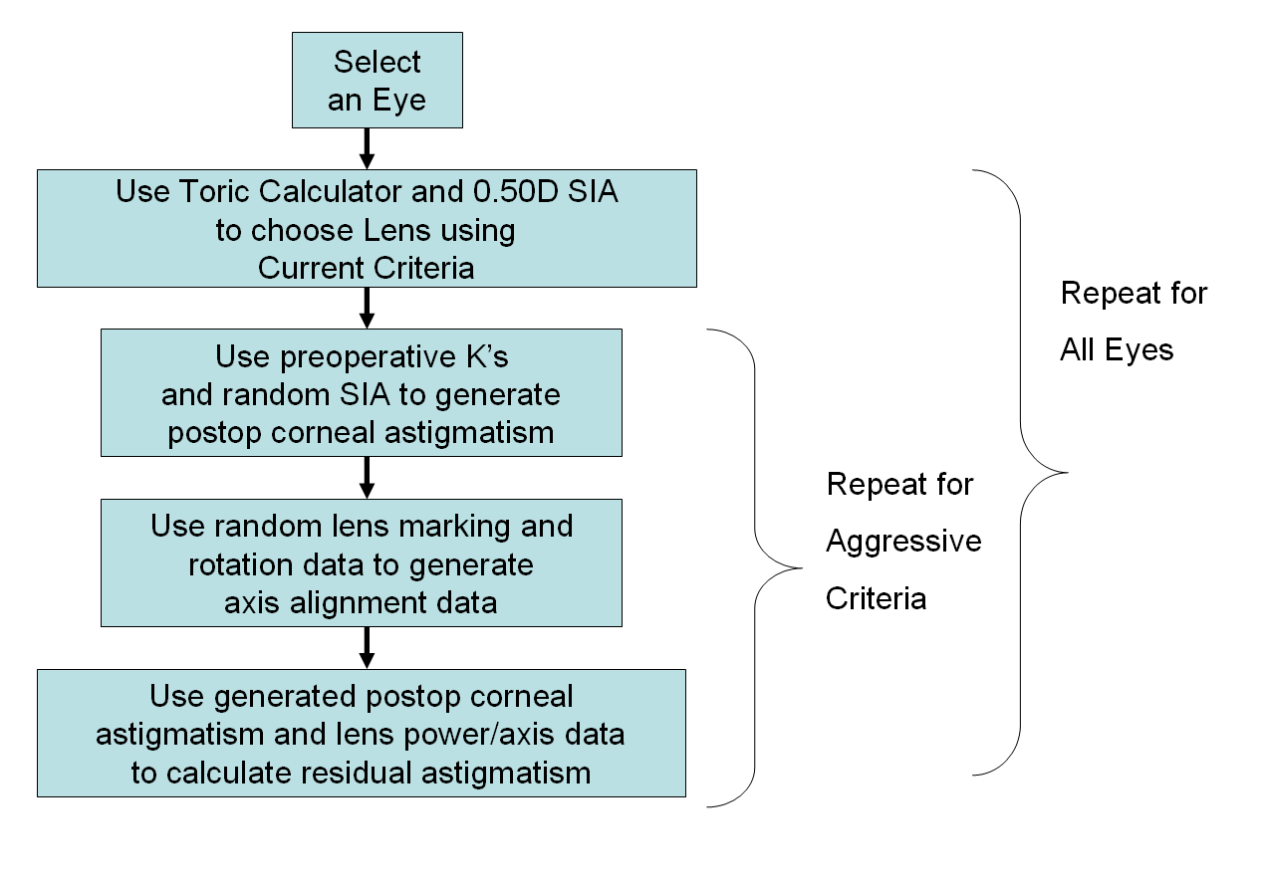
Flowchart of the Monte Carlo simulation of toric IOL surgery.

The Monte Carlo simulation was conducted as follows. A table of 2,000 random values of surgically induced astigmatism, lens marking error and lens rotation data were created from the simulation data above. A given eye and lens combination was then mathematically 'operated on.' The preoperative corneal astigmatism was modified by random surgically induced astigmatism and a lens chosen on the basis of the current or aggressive lens selection criteria. The lens choice was rotated away from the ideal orientation based on the estimated error in corneal marking and lens rotation. The resultant refractive astigmatism was calculated based on the vector sum of the resultant corneal astigmatism and toric lens astigmatic correction. This simulation was performed for both of the toric lens selection criteria. Figure 1 shows a flowchart of the procedure used.

The primary measure of interest was the change in the distribution of residual astigmatism (magnitude and axis) for the simulated patient population when the two lens assignment strategies above were implemented. Statistical testing with appropriate parametric and non-parametric tests was conducted using a significant p value of < 0.05.

## Results

**Figure 2:**
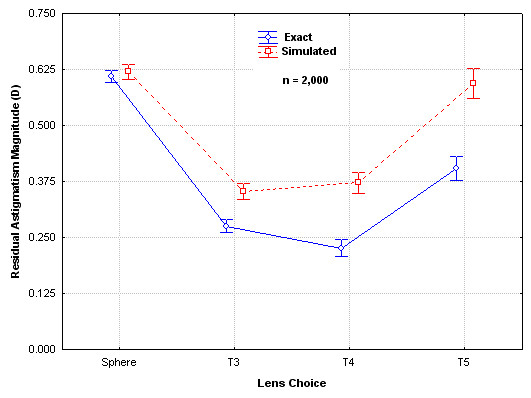
Residual Astigmatism Magnitude for Exact and Simulated Surgery.

Using the current toric lens selection criteria, the Monte Carlo simulation data can be compared to the results expected if the surgery had no variability. For purposes of this analysis the results calculated with no random error are termed "Exact", while those from the Monte Carlo simulation are termed "Simulated". Results are shown in Figure 2. Differences between the Exact and Simulated results are statistically significantly different for all lenses (ANOVA, p < 0.05).

Note that in all cases the residual astigmatism is higher with the Monte Carlo simulation, which is not surprising given the fact that the errors in induced astigmatism, corneal marking, lens placement and lens rotation all reduce the effect of the toric lens along the intended axis of correction. Note also that the differences grow larger with the increase in toric lens astigmatic power; the relative effect of lens axis alignment errors produces larger absolute errors. For the spherical lens the difference between exact and simulated is least because only the surgically induced astigmatism variable matters in this case – lens rotation and marking errors are of no consequence with a spherical lens.

**Figure 3:**
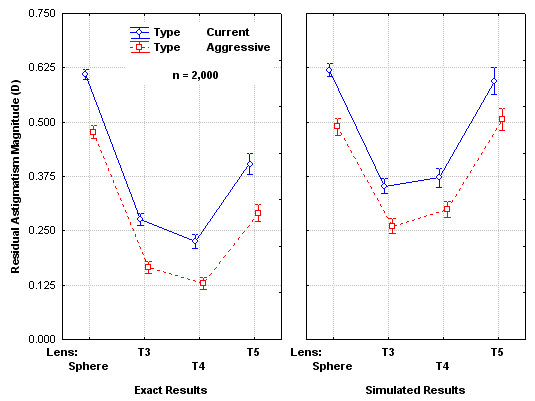
Residual Astigmatism Magnitude for Exact and Simulated Surgery using two Difference Lens Selection Criteria.

Figure 3 is an extension of Figure 2 to show both the Current and Aggressive toric lens selection criteria. Calculated residual astigmatism is statistically significantly different by toric lens, toric lens selection criteria and exact/simulation group (ANOVA, p < 0.05). Differences in simulated results are greatest in the Sphere category where the Aggressive toric lens selection criteria produce a significant reduction in the residual astigmatism. This is the result of assigning more eyes to astigmatic correction with the T3 lens under the Aggressive criteria.

It appears, then, that the Aggressive criteria will produce lower residual astigmatism in the population of eyes on average but these average differences are nominal. The more important considerations are the number of eyes in which a significant change occurs as a consequence of the two selection criteria for absolute magnitude and axis shift, and the number of eyes for which residual astigmatism is significant,

Table 2 contains a summary of the number of eyes for which lens selection changed based on the two criteria. This is the population of interest. Under the aggressive lens selection scenario 34% of previously spherical lenses were assigned to T3 lenses, 42% of T3 lenses were increased to T4 and 25% of T4 lenses were increased to T5. In all, 31% of eyes (629/2000) had a different lens selection under the aggressive criteria.

**Table 2:** Change in Lens Selection from Current to Aggressive Criteria

From Sphere	Eyes	To Sphere	T3	T4	T5
	787	**522**	265		
T3	622		**359**	263	
T4	398			**297**	101
T5	193				**193**
					
	Unchanged	1371	69%		
	Changed	629	31%		
	**Total**	**2000**			

**Figure 4:**
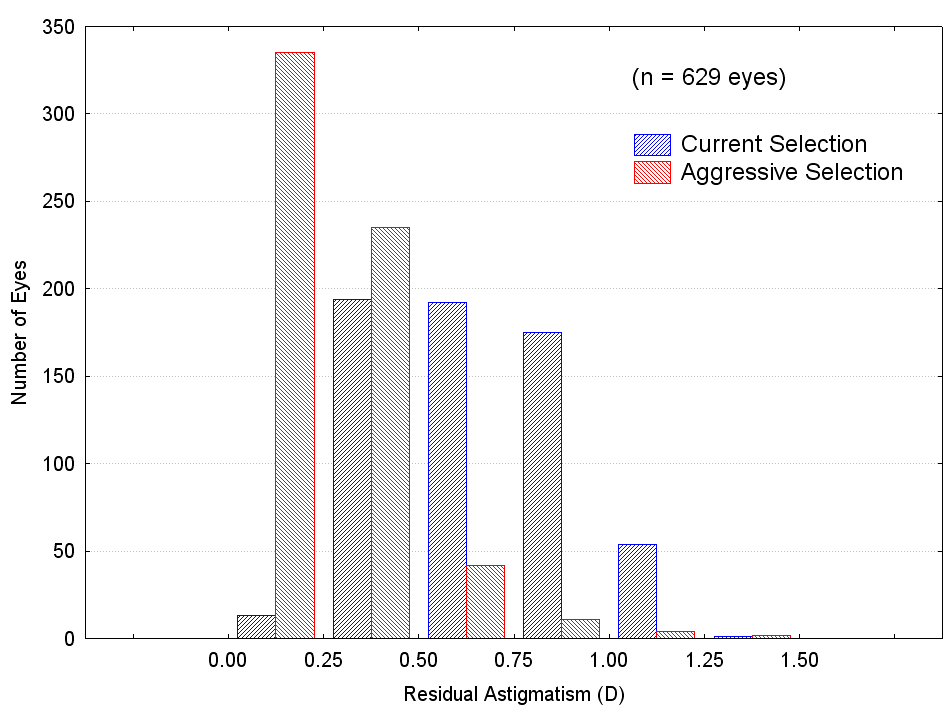
Residual Astigmatism Magnitude Distribution in Patients where Lens Selection Criteria Suggest a Different Lens.

The distribution of residual astigmatism for the patients with a lens change for the two lens selection scenarios is shown in Figure 4. It can be seen that there is a considerable shift to lower astigmatism values in the patients when the aggressive lens selection scenario is adopted. Mean expected residual astigmatism was 0.38D in the aggressive lens selection group. The simulated results suggest that 91% of patients in this group would have <= 0.50D of residual astigmatism vs. 33% if the current lens selection criteria were used. Only 1% of patients would have >1.00 D of residual astigmatism under the aggressive scenario vs. 9% under the current scenario. These are not

Table 3 shows where the changes in residual astigmatism occurred in the 629 eyes. The grey diagonal contains those eyes with the same degree of astigmatism under both lens selection criteria (the lens selected with either scenario did not change). Values to the bottom right are those with lower magnitudes under the aggressive selection criteria while values to the top right are those eyes with greater magnitudes. As can be seen, 60% of eyes have lower residual magnitude, 38% are unchanged and 2% have higher magnitude.

**Table 3:** Residual Astigmatism Distribution Under Two Lens Selection Criteria

	Aggressive Criteria					
Current Criteria	To 0.50D	0.50 < and <= 0.75	0.75 < and <= 1.00	1.00 < and <= 1.25	1.25 < and <= 1.50	Total
To 0.50D	202	5				207
0.50 < and <= 0.75	161	30	1			192
0.75 < and <= 1.00	157	7	9	2		175
1.00 < and <= 1.25	49		1	2	2	54
1.25 < and <= 1.50	1					1

Total	570	42	11	4	2	629

While these results related to astigmatism magnitude appear very good there is a directional component to astigmatism as well. With the degrees of freedom in this simulation it is problematic to determine how best to include consideration of angle, but the following approach was deemed reasonable.

**Figure 5:**
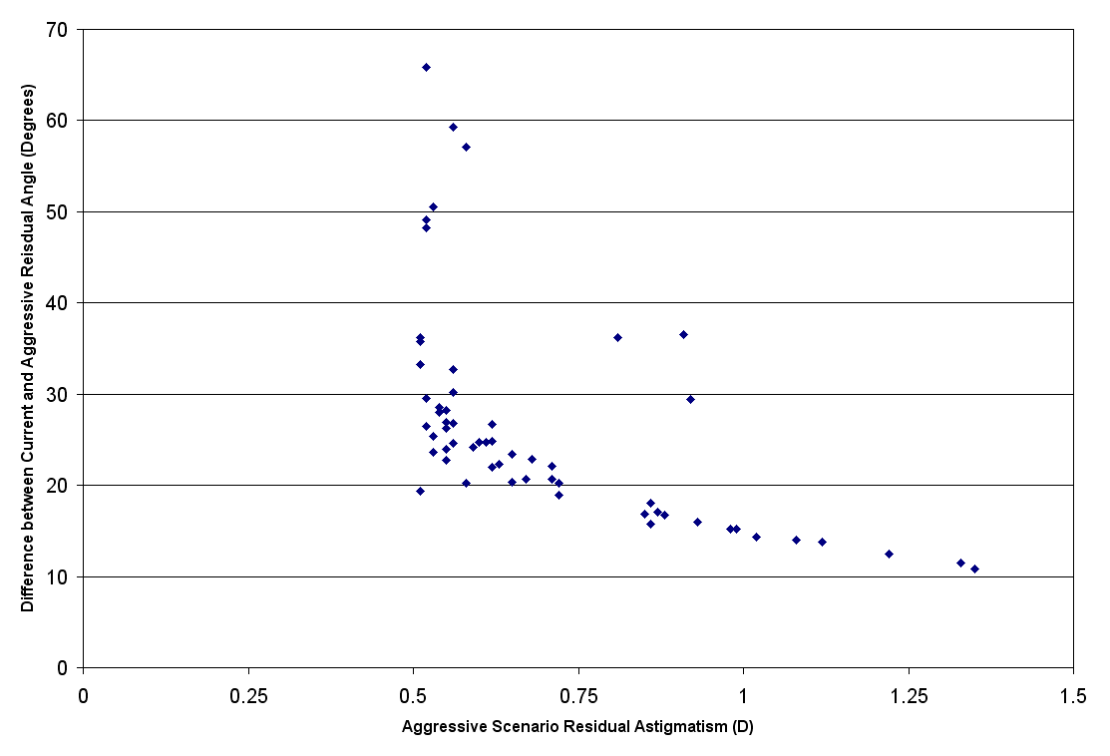
Aggressive Scenario Residual Astigmatism by Angle Difference Between Current and Aggressive Scenarios.

There are 59 eyes in Table 3 where the aggressive lens selection criteria indicate a residual astigmatism of > 0.50D. Vector mathematics can be used to determine how different the angles of these are from the corresponding angles associated with the current lens selection criteria. In other words, how much change in astigmatic axis did the aggressive lens selection criteria introduce? Figure 5 shows this astigmatic angle difference as a function of the residual astigmatism expected for the eyes with residual astigmatism > 0.50D. As can be seen, all changes in angle greater than 40 degrees are associated with < 0.625D of residual astigmatism. All residual astigmatism values > 1.00D have an associated angle difference of <15 degrees.

## Discussion

The analyses above provided the opportunity to predict the performance of the AcrySof^® ^Toric^® ^intraocular lens in simulated 'real-world' conditions, where surgical variables were allowed to vary and the effect on the surgical result could be calculated. One of the reasons for performing this analysis was to determine if the toric lens selection criteria originally suggested by Alcon were overly conservative. It can be seen in this analysis that the mean residual astigmatism with the current toric lens selection criteria is higher than with more aggressive criteria, but that the likelihood of overcorrection was minimal with these current criteria. In other words, to avoid overcorrection in a few individuals, the group had a slightly higher residual astigmatism than might have been possible with more aggressive lens selection.

The advantage of the Aggressive lens selection criteria is that, on average, patients would have significantly less residual astigmatism, but the likelihood of overcorrection increases; the fact that surgery is somewhat variable mitigates the latter. Using the Aggressive toric lens selection criteria from Table 1 it can be seen that a significant number of patients (31%) would have had a higher astigmatic correction suggested, with a significant reduction in their residual astigmatism. While some potential for overcorrection was evident, based on the change in astigmatic angle, high degrees of angle change were associated with low levels of astigmatism, suggesting that the issue of potential overcorrection is mitigated by surgical variability. The degree to which a low level of astigmatism is evident to the patient is not well-established but 0.50D is considered a nominal level [17,18]. Small levels of residual astigmatism can actually increase depth of focus slightly (a non-zero circle of Sturm) at the expense of maximum achievable acuity; patient preference in this regard is again not well-established.

The importance of this analysis is as follows. If, in review of clinical data, a surgeon noted that they had few cases of residual astigmatism near zero and no eyes with residual astigmatism at a significantly different axis than the preoperative corneal astigmatism, then there is a high likelihood that they could improve their results without major overcorrections. They may consider being more aggressive in their choice of AcrySof^® ^Toric lens power, or they may want to reevaluate their surgically induced astigmatism to check both the magnitude and the variability. Similarly, if they found that they had a significant number of patients with residual astigmatism > 0.75D who had a spherical IOL implanted, they might consider lowering the amount of corneal astigmatism they wanted to see preoperatively before considering a T3 lens. In the cases here, any eye with corneal astigmatism calculated as >0.75D from the AcrySof^® ^Toric^® ^calculator was provided a T3 lens and the simulation suggests no eye would have any significant residual astigmatism at a significantly different axis while a considerable percentage of eyes would have had a significant reduction in expected residual astigmatism.

In the above regard it is worth noting that Alcon's toric lens calculator has recently been adjusted to suggest a T3 lens when the combined corneal astigmatism and SIA exceed 0.90D, lower than the original value of 1.03D when the calculator was first introduced. No changes to other lens selection criteria have been made but future modifications may include calculating and showing the expected residual astigmatism when several different lenses are used, providing greater flexibility to the surgeon in toric lens planning.

Finally, it should be noted that this type of simulation can be used with the appropriate input variables to evaluate expected performance of other toric lenses or spherical lenses. Well-designed simulations can provide clinically-relevant data without the requirements related to a clinical trial.

## Conclusion

Monte Carlo simulation suggests that surgical variability and lens orientation/rotation variability may combine to produce small reductions in the correction achieved with the AcrySof^® ^Toric^® ^IOL. Adopting more aggressive lens selection criteria may yield significantly lower residual astigmatism values for many patients, with negligible overcorrections. Surgeons are encouraged to evaluate their AcrySof^® ^Toric^® ^outcomes to determine if they should modify their individual lens selection criteria, or their default surgically induced astigmatism value, to benefit their patients.

## Competing interests

WH acts as a consultant to Alcon Laboratories, Inc. in the area of intraocular lens mathematics.

RP is an employee of Alcon Laboratories, Inc.

## Authors' contributions

RP conceived of the simulation. WH provided the required clinical data. WH and RP reviewed the design, assigned the simulation variables, reviewed the results and wrote/reviewed the final paper.

## Pre-publication history

The pre-publication history for this paper can be accessed here:


